# Prevalence and Factors Associated with Iron Deficiency Anaemia among Children Aged 6-23 Months in Southwestern Uganda

**DOI:** 10.1155/2024/6663774

**Published:** 2024-03-04

**Authors:** Dickson Kajoba, Walufu Ivan Egesa, Solomon Muyombya, Yamile Arias Ortiz, Martin Nduwimana, Grace Ndeezi

**Affiliations:** ^1^Department of Paediatrics, Kampala International University, Kampala, Uganda; ^2^Department of Paediatrics, Nile International Hospital, Jinja, Uganda; ^3^Department of Statistics, Bugema University, Kampala, Uganda; ^4^Hispalense Institute of Paediatrics, Seville, Spain; ^5^Quirónsalud Campo de Gibraltar Hospital, Cadiz, Spain; ^6^Department of Paediatrics, Makerere University, Kampala, Uganda

## Abstract

Iron deficiency anaemia is still a global public health concern with the highest burden among children 6 to 23 months due to their rapid growth spurt exceeding breastmilk supply. Therefore, nutritional supply is a key source of iron to attain the required nutrients for better growth and development. This was a cross-sectional descriptive study done at Ishaka Adventist Hospital (IAH) and Kampala International University Teaching Hospital (KIUTH) from April to July 2022. Participants were consecutively enrolled in the study. Structured questionnaires, 24-hour dietary recall, and clinical assessment were used to obtain data. Data analysis was done using the statistical package for social scientists (SPSS) V22.0. Bivariable and multivariable analyses were done using logistic regression for associations with significance set at *P* value < 0.05. A total of 364 participants were enrolled, with the majority being males (198, 54.4%) and born at term (333, 91.5%). The modal age was 12-17 months [163(44.8%)] with a mean age of 14.1 months (SD 5.32). The overall prevalence of IDA was 151/364 (41.5%). The factors associated with IDA included male sex (aOR 1.61), current episode of diarrhoea (aOR 1.71), poor meal frequency (aOR 1.78), no vegetable consumption (aOR 2.47), and consuming fruits once (aOR 1.97) in 7 days preceding the study. The study finds a high prevalence of IDA among infants 6-23 months with at least four in 10 being affected. Screening for IDA should be recommended in male children with current diarrhoea, poor intake of fruits and vegetables, and poor meal frequency. The Mentzer index is an equally good alternative screening test for IDA.

## 1. Background

Iron deficiency (ID) is the commonest micronutrient deficiency worldwide. It affects over 42% of all children [1, 2] in both developed and developing countries. In Europe, ID prevalence ranges between 2% and 9%, while in the USA, it varies from 6.6% to 15.2% for ID and from 0.9% to 4.4% for iron deficiency anaemia (IDA) for children aged1-3 years [3]. The African region is greatly affected with about 60% of children under 5 years anaemic with 50% due to ID. Sub-Saharan Africa has a prevalence of 52% for ID with a range of 56.8%-62.5% [4, 5].

In Uganda, there has been a general decline in anaemia prevalence among children under 5 years from 64% in 2000 to 53% in 2016 [6]. Despite the decline, it is still ranked by the World Health Organization (WHO) as a severe public health concern and children less than 24 months are the most affected (greater than 70% prevalence) [7, 8].

IDA is caused by inadequate dietary intake in the face of rapid growth, highly marked nutrient loss, impaired absorption, or altered nutrient metabolism [9, 10]. It may cause permanent disruption in the mental and motor functioning of the children affected. These include learning difficulties, pseudotumor cerebri, sleep disturbance, sixth nerve palsy, breath-holding spells, deficit in attention, disorders in behaviour, reduction in perception, retardation in developmental tests both in mental and motor, and reduced auditory and visual functions. These effects can persist for over 12 years even after treatment [1, 11]. Prevention of premature delivery, exclusive breastfeeding during the first 6 months, provision of safe and iron-rich complementary foods from 6 months through 2 years, and avoiding whole cow's milk in the first 12 months are key strategies for the prevention of IDA [1, 12, 13].

Unfortunately, biomarkers for ID diagnosis like serum ferritin are not routinely available and affordable in developing countries [14]. RDW and Mentzer index are indices that have been identified as valid surrogates in the detection of IDA [15, 16]. RDW is a measure of the heterogenicity of red cell size currently generated by automated blood cell counters [17]. It is regarded as a cost-effective and sensitive test to differentiate iron deficiency anaemia from other types of microcytic anaemia if ferritin and iron studies are not available [10, 16]. Studies have found that when compared with serum ferritin, RDW in the presence of microcytosis had a sensitivity of 82.3%-99%, specificity of 69.4%-90%, positive predictive value of 73%-90.5%, negative predictive value of 73%-98.9%, and accuracy of 73%-75% [14, 18–21]. Its increase is considered the earliest haematological manifestation of IDA [14].

On another hand, the Mentzer index, which is the mean corpuscular volume divided by red cell count, has also been suggested as a valid test for IDA screening in developing countries with a value > 13 suggestive of IDA [15, 22, 23]. When compared with serum ferritin, it has a sensitivity of 93% and a specificity of 84%[15]. This study, therefore, determined the prevalence and factors associated with iron deficiency anaemia among children 6-23 months and compared the relationship between the Mentzer index and the combination of RDW in the presence of microcytosis in the diagnosis of IDA.

## 2. Methods and Materials

### 2.1. Study Area

The study was carried out at IAH and KIUTH located in Bushenyi-Ishaka municipality, Bushenyi district, Southwestern Uganda. IAH is a mission hospital under the Seventh-day Adventist Church while KIUTH is a private hospital owned by Kampala International University. Bushenyi-Ishaka municipality is situated 358 km west of Kampala, the capital city of Uganda. The majority of the indigenous residents are subsistence farmers and small-scale business owners.

### 2.2. Research Design and Methodology

The study was a hospital-based descriptive cross-sectional study using a quantitative study design. It was carried out from April to July 2022. Inclusion criteria were children 6-23 months and caregivers who consented to the study. Children who had received blood transfusion 3 months before the study and iron supplementation within a month prior to the study were excluded. The sample size was calculated using the Kish Leslie formula (1965), *N* = *P* (1 − *P*)/*d*2, where *N* is the sample size, *P* is the expected prevalence, and *d* is the margin of error. The sample size of 364 participants was based on the prevalence of 36.6% in Amuria, eastern Uganda [24]. Using consecutive enrolment, children 6-23 months were enrolled in the study from the respective outpatient and inpatient departments until the required sample size was obtained. Caregivers were given explanations about the study protocols, and informed consent was obtained. Age was confirmed using the child's date of birth, and for those who came with child health cards, the age was verified from the cards. A questionnaire was used to collect information about demographic information (age, sex, gestation, age at birth, weight, length, maternal age, and occupation), medical factors, dietary factors, and use of blood boosters, particularly iron supplements.

From each child, 2 milliliters of venous blood was collected into an EDTA-containing vacutainer after disinfecting the skin area with an alcohol swab. The sample was immediately transported to the lab where an automated coulter counter (BC 3000 Plus Mindray Auto Haematology Analyzer, Shenzhen, China) was used to perform a complete blood cell count (CBC).

Anaemia status was defined as haemoglobin (Hb) of less than 11.0 g/dl for ages 6 months to 59 months. It was classified as mild (10-10.9 g/dl), moderate (7.0-9.9 g/dl), and severe (<7.0 g/dl) [25, 26] as recommended by WHO. The Mentzer index was derived by dividing the mean cell volume by red cell blood count as obtained in CBC. A Mentzer index value of >13 in the presence of haemoglobin concentration less than 11 g/dl was suggestive of iron deficiency anaemia [23]. Additionally, microcytosis was determined by the mean corpuscular volume of <74 Fentoliters or on a peripheral film if the mean corpuscular volume (MCV) was normal or high [27, 28]. Thus, IDA was diagnosed as Hb < 11 g/dl, with RDW > 14 in the presence of microcytosis or Mentzer index > 13 with Hb < 11 g/dl. All tests were done from the KIUTH laboratory.

### 2.3. Ethics Approval and Consent of Participants

The research study followed the Declaration of Helsinki of 1964. Ethical approval was obtained from the Kampala International University-western campus research ethics committee with registration number KIU-2021-56. Institutional consent was sought, and informed consent for the infants was obtained from the parents/legal guardians before enrolment to the study.

### 2.4. Data Analysis

Descriptive analysis using frequencies and percentages was used to analyze independent and dependent variables using SPSS version 22.0. The association between independent and dependent variables was determined using binary logistic regression for both bivariable and multivariable analyses with *P* value significance set at <0.05. The relationship between the Mentzer index and RDW in the presence of microcytosis was established using the Spearman correlation coefficient. The test of significance was determined at a *P* value of 0.05.

## 3. Results

### 3.1. Study Participants

**Figure 1:**
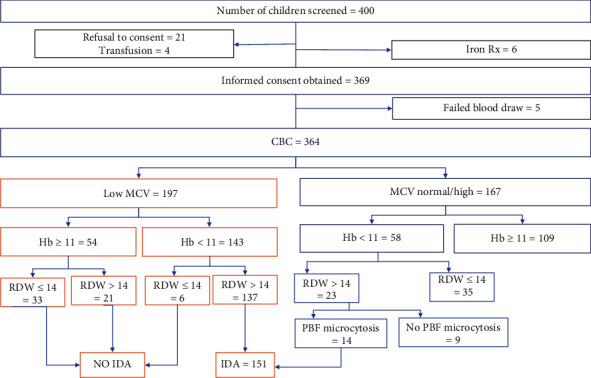
Study participants profile.

Children attending care at IAH and KIUTH were enrolled in the study between April and July 2022. The study profile is shown in Figure 1.

### 3.2. Baseline Characteristics

A total of 364 caregiver-child pairs were enrolled in the study. The majority of the participants were males (196, 53.8%) and born at term (333, 91.5%) with a birth weight of ≥2.5 kg (326, 89.6%). The modal age was 12-17 months of age (163, 44.8%) with a mean age of 14 months (skewness 0.048, SD 5.32), and the caregivers were mostly 21-30 years of age (248, 68.1%) with the highest level of education being tertiary (147, 40.4%) and (101, 27.7%) were formally employed. This is shown in Table 1.

### 3.3. Prevalence of Anaemia and Iron Deficiency Anaemia among Study Participants

Among the study participants, 55.2% (201/364) had anaemia with haemoglobin < 11 g/dl, and 44.5% (151/364) of participants had iron deficiency anaemia. Three-quarters (75.1% (151/201)) of anaemia was due to ID.

### 3.4. Severity of Anaemia and IDA among the Study Participants with Anaemia

Most participants with IDA had moderate anaemia (78, 51.7%), followed by those having mild anaemia (57, 37.7%), while those with non-IDA had a majority with mild anaemia (27, 54%). This is shown in Table 2.

### 3.5. Factors Associated with IDA

At bivariable analysis, male sex (cOR 1.65), vegetable consumption in the last 7 days (cOR 2.50), fruit in the 7 days preceding the study (cOR 1.81), acceptable meal frequency (cOR 1.38), and having a current episode of diarrhoea (cOR 1.68) were statistically associated with IDA. Poor dietary diversity was noted to increase the chances of having IDA by 1.076 times compared to those with good dietary diversity; however, it was not statistically significant. The factors that were statistically significant at a bivariable level of analysis were subjected to multivariable logistic regression. This is shown in Table 3.

At multivariable analysis, the male child had 61.2% (AOR 1.61 (1.03-2.53), P 0.04) increased risk for IDA compared to females and was significant.

Having a current episode of diarrhoea was associated with 1.71 odds for IDA (AOR 1.71 (1.03-2.82), P 0.04) compared to those without diarrhoea.

Not consuming vegetables and eating fruits once in 7 days before the study was associated with 2.47 times (AOR 2.47 (1.35-4.52), *P* < 0.01) and 1.97 times (AOR 1.97 (1.11-3.52) P 0.02) increased risk for IDA, respectively, compared to their counterparts' consumed vegetables or fruits more than 5 times in 7 days preceding the study.

Additionally, poor feeding frequency was associated with 78.1% higher odds (AOR 1.78 (1.14-2.77), P 0.01) for IDA compared to those having a good dietary frequency and was statistically significant as shown in Table 3.

### 3.6. Comparison of Mentzer Index with RDW in the Presence of Microcytosis in the Diagnosis of Iron Deficiency Anaemia

The prevalence of IDA using the Mentzer index was 45.3% compared to the 41.5% prevalence using RDW and microcytosis. Further analysis found a Spearman correlation coefficient of 0.521 (*P* < 0.001), suggesting a strong positive significant correlation between the two tests. This is summarised in Table 4.

## 4. Discussion

The overall prevalence of IDA among the study participants was 44.5%. Three-quarters (75.1%) of the anaemic participants were due to iron deficiency. This can be attributed to the methods used in the diagnosis of IDA. This study employed a combination of red cell distribution, width, and microcytosis (on complete blood count and peripheral film) to derive the diagnosis. RDW is a simple and widely available test in routine haematological tests that measures the degree of the RBC size heterogenicity [29]. It equally can express the smallest variation in red cell size which is accompanied by early iron deficiency unlike the other red cell indices (MCV, mean corpuscular haemoglobin (MCH) and mean corpuscular haemoglobin concentration (MCHC)) [30]. There is usually an overlap between patients with microcytic hypochromic anaemia in regard to red cell indices. This was observed in a study in Dhaka Shishu Hospital where there was no significant difference between IDA and thalassemia regarding MCV, MCH, and MCHC. A difference, however, was noted with RDW with statistical significance (*P* < 0.001) [31]. RDW is a very sensitive measure that changes earliest in the presence of iron deficiency anaemia even before microcytosis [1]. Thus, it is a sensitive measure in differentiating IDA from other causes of microcytic anaemia [1, 14, 32]. The peripheral film was considered as an earlier way for microcytosis before CBC mean corpuscular volume changes are observed. This further gives an earlier diagnosis of microcytic anaemia [28, 33]. It is thus a recommended test in the absence of iron studies for the diagnosis of IDA [31]. This could be one of the reasons why the study findings were lower than that observed in Lacor Hospital, Uganda, at 65.4% [25] and in Tanzania at 76% among children 6-23 months [34] where the diagnosis of IDA was based on peripheral film and Hb levels, respectively.

On another hand, the prevalence was higher than 17.7% in Eastern Uganda [24] and 21.1% in Entebbe [35]. The higher prevalence is explained by the fact that the age category used in the current study is highly prone to IDA due to its rapid growth and accelerated nutrient demand [36]. In addition, a wide age category was used compared to the Eastern Uganda study that enrolled children aged 11-23 months and both studies used iron studies for the diagnosis of IDA which is a more specific test compared to RDW plus microcytosis. Unfortunately, iron studies are costly and not readily available. The study findings also agree with other studies that IDA is the leading cause of anaemia among children [1, 2]. This anaemia burden is still ranked as a severe public health concern [26] and thus an area of great concern to the public.

The majority of the study participants with IDA had mild-moderate anaemia (135, 89.4%) with the commonest being moderately anaemic (78, 51.7%), and only 16 (10.6%) had severe anaemia. This is because mild to moderate IDA is asymptomatic, and therefore those affected do not seek health attention. This agrees with a study in Korea where the prevalence was 36.9% (*N* = 491), 59.3% (*N* = 789), and 3.8% (*N* = 50) for mild, moderate, and severe anaemia, respectively [37].

Male infants had more than 60% higher odds (AOR 1.61 (1.03-2.53), P 0.04) of having IDA compared to females. This could be explained by the fact that compared to girls, boys have a higher pre- and postnatal growth rate but with low iron storage states due to the observed larger intestinal iron loss, lower absorption, and more frequent infections [37–41]. The study findings agree with a study in Finland where iron deficiency and IDA were more prevalent in boys compared to girls (19% vs. 9%) [42]. It also concurs with a study in western Kenya where male children had an increased risk for ID compared to females [43] and in Pakistan, being female was associated with reduced odds for IDA [38].

Children with current diarrhoea episodes were 1.7 times more likely to have IDA compared to those without diarrhoea. This is because diarrhoea interferes with iron absorption due to the short transit time of feeds and damage to the microvilli responsible for iron absorption [41]. The study findings agree with a study in Indonesia where current diarrhoea was 1.2 times more likely to be associated with IDA compared to those without diarrhoea [44]. Additionally, this study concurs with a study in Nepal where one in every 3 children with acute diarrhoea had depleted iron stores and 31.0% of the anaemia observed among the study participants was due to iron deficiency [45]. Whereas studies have shown that diarrhoea is associated with IDA due to reduced absorption, it is also possible that diarrhoea can occur as a result of IDA. This is because iron deficiency is associated with reduced immunity leading to recurrent infections including those of the gastrointestinal tract [46].

Children with poor acceptable meal frequency were 1.8 times more likely to develop IDA compared to their counterparts that had an acceptable meal frequency. This is because poor meal frequency limits the iron provision from complementary feeds to cater to the accelerated iron demand for the fast-growing infant. Additionally, reduced meal frequency renders the child more dependent on breastmilk which is not sufficient to supply the required nutrients including iron above 6 months of age [47, 48]. The study findings concur with a study in northern Uganda where a lack of complementary feeding and insufficient complementary feeding increased the risk of IDA by four times and two times, respectively [25]. Additionally, the study agrees with a study in Kenya where low diet intake was observed as a major cause of anaemia in children aged 6 months to 14 years [49].

Children who never ate vegetables in 7 days before the study were 2.5 times more likely to have IDA, while consuming fruits once in 7 days before the study increased the likelihood of developing IDA by more than 90% compared to those that consumed vegetables and fruits more than 5 times in 7 days before the study, respectively. This is because vegetables and fruits contain iron and vitamin C which play a crucial role in enhancing iron absorption from nonhaem iron in the cereal-based diet which predominates the staple foods in the region [50, 51]. Plant sources are highly rich in iron, but iron is not readily bioavailable as only 5-15% is available for body absorption [50]. One of the reasons for the low uptake of vegetables among children is the misconception that it is not acceptable to feed children vegetables and not palatable to children in the face of more palatable foods [52]. Another negative impact of vegetables is the high content of phytates and phenolic compounds which inhibit iron absorption in the gut. Thus, considering a particular food item may be misleading, but rather a complete meal philosophy is more inclusive, especially balancing between enhancement (ascorbic acid, heme, iron, and fermented foods) and inhibition [50]. This study's findings agree with a study in South Africa where consumption of “vegetables and fruits other than vitamin A-rich” was associated with lower odds of being anaemic at both 24-hour recall and 3-day recall [53]. Furthermore, it concurs with a study in Tanzania and Brazil where not consuming vegetables and consumption of fruits/fruit juice < 2 portions/day increased the risk for IDA by two times [54].

Accordingly, WHO recommends special attention to foods that enhance iron absorption/consumption of fruit and vegetables as a way to mitigate the burden of IDA among children the world over [32].

Worth noting in this study is that there was no association between meat consumption and IDA among the study participants though it is a known source of nutritional iron. This could be attributed to the fact that the majority of the participants at least consumed meat once a week (67.3%). Additionally, iron level could be contributed through the use of Bushera which is a key feed during complementary feeding as porridge [55]. Bushera made out of millet is estimated to contain about 2-8 mg/100 g of bioavailable iron and it is associated with a 13.2% increment in HB following regular consumption as porridge/meal for at least 21 days compared to other regular diets and a 54.7% increase in serum ferritin levels [56].

The study found a strong positive significant correlation between RDW in the presence of microcytosis and Mentzer index in the diagnosis of iron deficiency anaemia (Spearman's correlation coefficient 0.521, *P* value < 0.001). This implies that both tests are very likely to give the same result when used independently. The study findings of a strong correlation between RDW and Mentzer index are supported by a meta-analysis comparing numerous red cell indices in differentiating IDA and beta thalassemia found a sensitivity of 1.0 (0.5-1.4) at 95% confidence interval, *P* value false positive rate of 0.001-0.5 (-0.9 to -0.1), and a *P* value of < 0.001 using RDW as reference [57]. Additionally, a study comparing RDW and Mentzer index found a sensitivity of 94.2% and 99.4% and PPV of 98.8% and 97.7% for the diagnosis of IDA when compared with serum ferritin among primigravids in Pakistan [21]. During iron deficiency, smaller RBCs are produced by the bone marrow resulting in a smaller RBC count and low MCV, thus a Mentzer index of >13 [58]. Therefore, RDW and Mentzer index can be used for early presumptive diagnosis of IDA and thus early initiation of treatment to avert the complications and long-term sequelae associated with IDA.

### 4.1. Limitations

The gold standard of serum ferritin for IDA was not used. However, the researcher diagnosed IDA based on RDW in the presence of microcytosis (low MCV and peripheral blood film smear).

## 5. Conclusions and Recommendations

IDA is highly prevalent among infants aged 6-23 months with at least four in 10 being affected. Screening for IDA should be recommended in male children with current diarrhoea, poor intake of fruits and vegetables, and poor meal frequency. The Mentzer index is an equally good alternative screening test for IDA.

## Figures and Tables

**Table 1:** Sociodemographic characteristics of children aged 6-23 months and their caregivers attending KIUTH and IAH, Bushenyi-Ishaka municipality.

Variables	Frequency (%)	Percentage
Age		
6-11 months	113	31.0
12-17 months	163	44.8
18-23 months	88	24.2
Sex		
Female	168	46.2
Male	196	53.8
Gestation age at birth		
Premature	31	8.5
Term	333	91.5
Birth weight		
<2.5 kg	38	10.4
≥2.5 kg	326	89.6
Maternal age		
≤20	14	3.8
21-30	248	68.1
31-40	90	24.7
>40	12	3.3
Maternal level of education		
At most primary	111	30.5
Secondary	106	29.1
Tertiary	147	40.4
Maternal occupation		
Housewife	79	21.7
Formal employment	101	27.7
Business	77	21.2
Peasant	107	29.4

**Table 2:** The severity of anaemia and iron deficiency anaemia among study participants.

Severity	IDA	Non-IDA (%)	Total (%), *N* = 201
Mild	57 (37.7)	27 (54)	84 (41.8)
Moderate	78 (51.7)	13 (26)	91 (45.3)
Severe	16 (10.6)	10 (20)	26 (12.9)

**Table 3:** Bivariable and multivariable logistic regression analyses for associated factors.

Variable	IDA, frequency *n* (%)		cOR (95% CI)	*P* value	AOR (95% CI)	*P* value
	No (%)	Yes (%)				
Age (months)						
6-11	68 (31.9)	45 (29.8)	1.22 (0.68-2.17)	0.51		
12-17	88 (41.3)	75 (49.7)	1.57 (0.92-2.68)	0.10		
18-23	57 (26.8)	31 (20.5)	Ref			
Sex						
Female	108 (50.7)	58 (38.4)	Ref		Ref	
Male	105 (49.3)	93 (61.6)	1.65 (1.08-2.52)	0.02	1.61 (1.03-2.53)	0.04
Gestation age (at birth)						
Premature	15 (7.0)	16 (10.6)	Ref			
Term	198 (93.0)	135 (89.4)	0.64 (0.31-1.34)	0.23		
Birth weight (kg)						
<2.5	19 (8.9)	19 (12.6)	Ref			
*≥2.5*	194 (91.1)	132 (87.4)	0.68 (0.35-1.33)	0.26		
Maternal age (years)						
≤20	11 (5.2)	3 (2.0)	0.38 (0.07-2.13)	0.27		
21-30	138 (64.8)	110 (72.8)	1.12 (0.35-3.61)	0.86		
31-40	57 (26.8)	33 (21.9)	0.81 (0.24-2.76)	0.74		
>40	7 (3.3)	5 (3.3)	Ref			
Maternal level of education						
Primary	67 (31.5)	44 (29.1)	1.01 (0.61-1.67)	0.98		
Secondary	57 (26.8)	49 (32.5)	1.32 (0.80-2.19)	0.28		
Tertiary	89 (41.8)	58 (38.4)	Ref			
Maternal occupation						
Housewife	42 (19.7)	37 (24.5)	1.36 (0.76-2.47)	0.30		
Formal employment	64 (30.0)	37 (24.5)	0.90 (0.51-1.57)	0.70		
Business	42 (19.7)	35 (23.2)	1.29 (0.71-2.33)	0.40		
Peasant	65 (30.5)	42 (27.8)	Ref			
Stunted^∗^						
No	149 (70.0)	110 (72.8)	Ref			
Yes	64 (30.0)	41 (27.2)	1.09 (0.82-1.44)	0.55		
Wasted^∗^						
No	173 (81.2)	120 (79.5)	Ref			
Yes	40 (18.8)	31 (20.5)	1.12 (0.66-1.89)	0.68		
HIV status						
Negative	209 (98.1)	143 (94.7)	Ref			
Positive	1 (0.5)	3 (2.0)	2.44 (0.57-10.35)	0.23		
Sero-exposed	3 (1.4)	5 (3.3)	4.39 (0.45-42.57)	0.20		
Pneumonia						
No	171 (80.3)	119 (78.8)	Ref			
Yes	42 (19.7)	32 (21.2)	1.10 (0.65-1.83)	0.73		
Current fever						
No	138 (64.8)	89 (58.9)	Ref			
Yes	75 (35.2)	62 (41.1)	1.28 (0.84-1.97)	0.26		
History of fever in last 2 weeks						
No	108 (50.7)	70 (46.4)	Ref			
Yes	105 (49.3)	81 (53.6)	1.19 (0.78-1.81)	0.41		
Current diarrhoea episode						
No	169 (79.3)	105 (69.5)	Ref		Ref	
Yes	44 (20.7)	46 (30.5)	1.68 (1.04-2.72)	0.03	1.71 (1.03-2.82)	0.04
Diarrhoea history in last 2 weeks						
No	149 (70.0)	114 (75.5)	Ref			
Yes	64 (30.0)	37 (24.5)	0.76 (0.47-1.21)	0.24		
Deworming in last 3 months						
No	79 (46.7)	59 (48.8)	0.92 (0.58-1.47)	0.74		
Yes	90 (53.3)	62 (51.2)	Ref			
Exclusive breastfeeding						
≤6 months	194 (91.1)	142 (94.0)	0.65 (0.28-1.47)	0.30		
>6 months	19 (8.9)	9 (6.0)	Ref			
Dietary diversity						
Poor	135 (63.4)	100 (66.2)	1.08 (0.83-1.40)	0.58		
Good	78 (36.6)	51 (33.8)	Ref			
Acceptable meal frequency						
No	89 (41.8)	84 (55.6)	1.38 (1.08-1.77)	0.01	1.78 (1.14-2.77)	0.01
Yes	124 (58.2)	67 (44.4)	Ref		Ref	
Dietary intake in the last 7 days						
Meat						
Never	71 (33.3)	48 (31.8)	0.68 (0.21-2.22)	0.52		
Once	61 (28.6)	48 (31.8)	0.79 (0.24-2.60)	0.69		
2-5 times	75 (35.2)	49 (32.5)	0.65 (0.20-2.14)	0.48		
>5 times	6 (2.8)	6 (4.0)	Ref			
Fish						
Never	132 (62.0)	98 (64.9)	2.04 (0.63-6.60)	0.23		
Once	45 (21.1)	30 (19.9)	1.83 (0.53-6.30)	0.34		
2-5 times	25 (11.7)	19 (12.6)	2.09 (0.58-7.60)	0.26		
>5 times	11 (5.2)	4 (2.6)	Ref			
Vegetables						
Never	40 (18.8)	50 (33.1)	2.50 (1.44-4.35)	<0.01	2.47 (1.35-4.52)	<0.01
Once	64 (30.0)	44 (29.1)	1.38 (0.81-2.34)	0.24	1.41 (0.80-2.47)	0.23
2-5 times	23 (10.8)	14 (9.3)	1.22 (0.57-2.60)	0.61	1.15 (0.52-2.55)	0.73
>5 times	86 (40.4)	43 (28.5)	Ref		Ref	
Chicken						
Never	170 (79.8)	111 (73.5)	3.92 (0.47-32.99)	0.21		
Once	24 (11.3)	34 (22.5)	8.50 (0.96-75.23)	0.05		
2-5 times	13 (6.1)	5 (3.3)	2.31 (0.22-24.32)	0.49		
>5 times	6 (2.8)	1 (0.7)	Ref			
Fruits						
Never	49 (23.0)	37 (24.5)	1.46 (0.80-2.69)	0.22	1.10 (0.57-2.14)	0.78
Once	75 (35.2)	70 (46.4)	1.81 (1.05-3.11)	0.03	1.97 (1.11-3.52)	0.02
2-5 times	29 (13.6)	13 (8.6)	0.87 (0.40-1.90)	0.72	0.92 (0.40-2.07)	0.83
>5 times	60 (28.2)	31 (20.5)	Ref		Ref	

^∗^Based on WHO child growth standards.

**Table 4:** A comparative table for the Mentzer index versus RDW plus microcytosis in the diagnosis of iron deficiency anaemia among the study participants.

Diagnostic test	Prevalence of IDA	
Hb < 11 g/dl + RDW > 14 + microcytosis	41.5%	
Hb < 11 g/dl + Mentzer index > 13	45.3%	
Spearman correlation coefficient		0.521
*P* value		<0.001

## Data Availability

Data is available from the corresponding author upon request.
